# Predictors of stunting among children aged 6-59 months in Kitui County, Kenya

**DOI:** 10.4314/ahs.v24i4.52

**Published:** 2024-12

**Authors:** Morris K Chui, Justus O Osero, Judy W Mugo

**Affiliations:** 1 Department of Family Medicine, Community Health and Epidemiology, School of Health Sciences, Kenyatta University, P.O.Box 43844-00100 Nairobi, Kenya; 2 Department of Family Medicine, Community Health and Epidemiology, Kenyatta University, P.O.Box 43844-00100 Nairobi, Kenya

**Keywords:** Stunting, predictors, children

## Abstract

**Background:**

Stunting in children below the age of five is a significant public health problem in Kenya. Stunting has transitory and lifelong effects on people and communities, including high risk of morbidity and death, lowered mental and physical growth, and decreased productivity.

**Objective:**

To investigate the predictors of stunting in children aged 6 to 59 months in Kitui County, Kenya.

**Methods:**

This was a cross-sectional analytical survey that used multistage cluster sampling. Data were gathered through questionnaires, interviews with key informants and discussions with focus groups. Anthropometric measurements were taken to assess the rate of stunting.

**Results:**

The stunting rate was 26.6%. The independent predictors of stunting included birth weight < 2500 grams (AOR = 2.302; 95% CI: 1.027-6.150; p: 0.043), having secondary education (AOR = 5.404; 95% CI: 1.607-18.173; p: 0.006), mother's MUAC < 23 cm (AOR = 2.845; 95% CI: 1.125-7.192; p:0.012), and having no access to a latrine/toilet (AOR = 0.008; 95% CI: 0.009-0.561; p: 0.013).

**Conclusions:**

Stunting was high and was influenced by sociodemographic factors, and the household environment.

## Introduction

Stunting in childhood remains among the most prominent hindrances to human progress worldwide. Stunting is linked to severe irreparable physical and neurocognitive impairment that undermines human development. This led to stunting being recognized as a critical health priority for the world and is at the center of global focus at the top levels with ambitious goals to be met by 2025^1^. Globally, about 22.0% of the world's children were stunted in 2020. Stunting in children below five years is decreasing in all parts of the world except in Africa where numbers are growing and with large differences in progress sub-nationally^2^. Eastern Africa has high stunting levels at 32.6%^3^. The Kenya Demographic and Health Survey of 2014 showed that 26% of children had stunting. At the county level, Kitui County had one of the highest proportions of stunted children in Kenya at 45.8%. Reviewed literature indicates that stunting is related to a wide range of factors. To begin with, stunting has been linked to caregiver's knowledge of a child's linear growth^4^,^5^. Stunting also correlates with a number of sociodemographic factors, including child age and sex^6^,^7^,^8^,^9^, maternal education^7^,^10^ household income^11^,^12^,^13^, number of children below 5 years in a family^14^,^9^ among others. Child feeding practices are strongly associated with stunting^15^,^10^. Moreover, morbidity poses higher risk of growth failure since infections lead to increased energy needs and expenditure, reduced appetite, loss of nutrients and nutrient malabsorption^16^.

Data exists on the correlation between environmental conditions and stunting among children. The use of safe water, handwashing with soap, and other sanitary practices reduce the risk of diarrhea and related illnesses that may significantly affect child growth^9^. There was little information on factors related to the high stunting prevalence in Kitui County, and no survey had been conducted focusing on stunting and its determinants. By gathering data on predictors of stunting specific to the study area, this survey helps in filling the knowledge gap and aids in formulating effective strategies to combat stunting.

## Methods

### Study design and setting

This was a cross-sectional analytical study at the community level that utilized quantitative and qualitative data collection techniques. The study was conducted in Kitui Central Sub-county, one of the eight sub-counties of Kitui County, located in the eastern part of Kenya.

### Study population

The survey targeted children between the ages of 6 and 59 months in Kitui Central Sub-County. The study population comprised of caregivers with their children, mothers of children who attended the focus group discussions, and key informants. The study included children aged 6 to 59 months and their caregivers who gave informed consent, and excluded children aged 6 to 59 months whose mothers were mentally impaired.

### Sample size

Sample size calculation utilized the Fisher et al. (1998) formula. The assumed prevalence of stunting in Kitui County was 45.8% according to the 2014 KDHS report. The 95% confidence level was used, and the margin of error set at 0.05. The minimum sample size was 382. However, a sample size of 420 was used taking into account a 10% non-response rate.

### Sampling technique

Multistage cluster sampling was used. Kitui County was purposively chosen due to its high stunting rate. Kitui Central Sub-County was also purposively selected owing to its cosmopolitan nature and a varied population comprising both urban and rural settlements. The third stage involved the selection of clusters from all the five wards of Kitui Central Sub-County based on probability proportional to population size. Two villages were picked per ward using simple random sampling. The last stage involved selecting households within the selected villages from a list of households with children aged 6 to 59 months. If the selected household had more than one eligible child, only one was randomly picked for the study.

### Research instruments

A structured data collection tool with closed-ended questions was used to gather quantitative data. This questionnaire also captured measurements of height/length and the mid-upper arm circumference of mothers. Additionally, an interview guide for key informants and a discussion guide for focus groups were used to obtain qualitative data. The data collection tools were written in English and administered in Kiswahili.

### Validity and reliability of the instruments

To assure validity, the survey instruments were reviewed by two university supervisors, and the researcher selected a large sample and randomly sampled the study participants. To ensure reliability, research assistants were thoroughly trained to administer the questionnaire, which was also pretested to ascertain its suitability.

### Pretesting

Research assistants participated in a pretest as part of training before starting data collection. After the pretest, adjustments were made to the research instruments to ensure that all questions were well constructed and well understood by the interviewers and the interviewees.

### Data collection techniques

Data were obtained from caregivers through interviews using validated structured questionnaires. The questionnaires gathered data on caregiver's knowledge of stunting, sociodemographic factors, child care practices, and environmental factors. Data on age and sex were obtained from the child's birth certificate or immunization card. The height or length of all children between 6 to 59 months was taken to assess stunting. Both height and length were measured to the nearest 0.1 cm. The MUAC of mothers was taken to assess their nutritional status.

### Data analysis

Survey data were analyzed with SPSS version 27. Anthropometric data were analyzed with the ENA for SMART software Jan 11th, 2020 version.

#### Logistic regression and model fitting

All the study variables were subjected to the binary logistic regression analyses to calculate the Odds Ratio (OR) for each independent variable. Before performing the multivariable logistic regression analysis, multicollinearity among the explanatory variables was assessed using the variance inflation factors and the Spearman correlation matrix. These tests showed that none of the variables were highly correlated, so all the predictors were included in the model. Variables that were statistically associated with stunting in the bivariate logistic regression analyses were added into a multivariable logistic regression model to adjust for potential confounding and estimate the adjusted Odds Ratios (AOR) in order to determine the independent predictors of stunting using the equation described below:


$$\text{logit}(\text{Y}=1)=\text{ln}\;\;\frac{\text{pr}(???=1)}{1-\text{pr}(???=1)}=\text{ln}\;\text{odds}(\text{Y}=1)={\text{b}}_{0}+{\text{b}}_{1}{\text{X}}_{1}+?\;{\text{b}}_{\text{k}}{\text{X}}_{\text{k}}$$

The statistical significance level was at p<0.05. Various tests were used to evaluate if the model fit the data. The chi-square test (likelihood ratio) indicated that the model is a significant improvement in fit compared to the intercept-only model, χ^2^(5) = 38.999, p=0.000. Additionally, the Hosmer and Lemeshow test was not statistically significant which indicated that the model was a good fit to the data [χ^2^(4) = 1.949, p=0.745]. In the model, the overall classification of cases to the group membership for the dependent variable was 75.4%. Qualitative information was transcribed and translated into English. Transcripts of the interviews were analyzed manually by coding, summarizing, categorizing, using direct quotes, and comparisons. Emerging themes were identified and triangulated with quantitative data.

### Ethical considerations

Ethical approval was granted by the Ethics Review Committee of Kenyatta University (application approval number PKU/2151/1295). The National Commission for Science, Technology, and Innovation provided a license authorizing the study. Consultations with County officials and community leaders were held to seek permission to conduct the research. Caregivers of children 6 to 59 months old in the chosen households were invited to give informed consent and to sign a form after the introduction of the survey by the research teams.

## Results

### Socio-demographic characteristics of the respondents

A total of 398 caregivers who had a child 6 to 59 months participated in the survey. Mothers ranged in age from 17-46 years, with a mean of 28.29 (± 5.780) years. The majority of women were married (79.4%), 38.4% had primary education, 42.7% had secondary education, and 18.8 percent had college or university education. Most of the mothers (66.8%) were self-employed. In the sample, 50.5% of the children were female and 49.5% were male. The children's mean age was 30.3 (± 15.1) months. Most children (87.4%) were delivered with normal birthweight of ≥ 2500 grams. More than half of the households (56.3%), reported an income of less than Kshs. 10,000. The average household size was 5 (±2) people, and almost three-quarters (72.1%) of the households had only one child aged below five years. Interviews held with key informants indicated that sociodemographic factors influenced stunting in Kitui County in several ways. For instance, the Sub-county Nutrition Officer explained: “*Poverty levels in Kitui are high at around 60% and many families have little income they can use to get enough food for their children”*. The County Public Health Officer commented: “*Teenage pregnancies are common and these girls want to go back to school, so they interrupt exclusive breastfeeding and the child is not given adequate care”*.

### Socio-demographic factors associated with stunting

As indicated in table 1, children weighing less than 2500 gm at birth had 2.5 greater odds of being stunted compared to children with a birth weight of 2500 gm or more. Children born to women with secondary education had 3.1times highelikelihood of getting stunted than children whose mothers had post-secondary education. Children whose mothers MUAC was < 23 cm had 3.1 greater odds of stunting than babies whose mothers had a MUAC ≥ 23 cm. Children from households with income below Kshs 10,000 had a 5-fold bigger risk of getting stunted than children from families whose income was Kshs 20,000 or more. Families with more than one child below the age of five had 1.7 greater odds of stunting in comparison to households that had only one child less than five years.

**Table 1:** Logistic regression of the association between sociodemographic factors and stunting

Variable	Stunted HAZ<-2SD n (%)	Not stunted HAZ≥-2SD n (%)	Crude Odds Ratio (95% CI)	p-value
**Child's age in months (N=398)**				
6-11	12 (24.0)	38 (76.0)	1.00 (reference)	
12-23	27 (25.5)	79 (74.5)	1.082 (0.495-2.367)	0.843
24-35	32 (33.0)	65 (67.0)	1.559 (0.718-3.383)	0.261
36-47	16 (19.5)	66 (80.5)	0.768 (0.329-1.793)	0.541
48-59	19 (30.2)	44 (69.8)	1.367 (0.589-3.177)	0.467
**Child's gender (N=398)**				
Female	50 (24.9)	151 (75.1)	1.00 (reference)	
Male	56 (28.4)	141 (71.6)	1.199 (0.769-1.872)	0.423
**Child's birth weight (N=398)**				
<2500gms	22 (44.0)	28 (56.0)	2.469 (1.342-4.545)	0.004*
≥2500gms	87 (24.1)	264 (75.9)	1.00 (reference)	
**Mothers age at childbirth (N=398)**				
15-19	11 (32.4)	23 (67.6)	1.222 (0.426-3.505)	0.709
20-24	36 (24.7)	110 (75.3)	0.836 (0.355-1.972)	0.683
25-29	33 (28.2)	84 (71.8)	1.004 (0.421-2.395)	0.993
30-34	17 (24.6)	52 (75.4)	0.835 (0.325-2.150)	0.709
≥35	9 (28.1)	23 (71.9)	Reference	
**Mother's education level (N=398)**				
Secondary	97 (30.0)	226 (70.0)	3.147 (1.508-6.570)	0.002*
Post-secondary	9 (12.0)	66 (88.0)	Reference	
**Maternal occupation (N=398)**				
Unemployed	22 (27.2)	59 (72.8)	1.356 (0.593-3.102)	0.741
Self employed	73 (27.4)	193 (72.6)	1.375 (0.670-2.8240	0.385
Employed	11 (21.6)	40 (78.4)	Reference	
**Mother's nutrition status (N=293)**				
<23 cm	11 (50.0)	11 (50.0)	3.106 (1.288-7.493)	0.012*
≥23cm	66 (24.4)	205 (75.6)	Reference	
**Household income in Kshs (N=398)**				
<10,000	73 (32.6)	151 (67.4)	5.125 (1.965-13.364)	0.001*
10,000-19,000	28 (24.1)	88 (75.9)	3.373 (1.227-9.267)	0.018*
≥20,000	5 (8.6)	53 (91.4)	Reference	
**No. of people in HH (N=398)**				
<5	45 (23.6)	146 (76.4)	Reference	
≥5	61 (29.5)	146 (70.5)	1.356 (0.866-2.122)	0.184
**Children under 5 in HH (N=398)**				
1	68 (23.7)	219 (76.3)	Reference	
>1	38 (34.2)	73 (65.8)	1.676 (1.040-2.702)	0.034*

* **Significant association between variables (p-value < 0.05)**

### Prevalence of stunting

The stunting rate was 26.6% (21.4-32.6 95% C.I.) and severe stunting was 3.8% (2.6-5.5 95% C.I.) as displayed in table 2 below. Boys had slightly higher prevalence (28.4%) when compared to girls (24.9%). In the FGDs, mothers were asked if stunting was a problem in their community. One mother responded: “*Yes, it is a big problem because there are many children who are short”*.

**Table 2:** Prevalence of stunting in children aged between 6 and 59 months

Stunting (Height-for-age z scores)	Frequency (N=398)	Percent
**Overall stunting (<-2 z scores)**	**106**	**26.6**
Moderate stunting (<-2 to ≥-3 z scores)	91	22.9
Severe stunting (<-3 z scores)	15	3.8
Normal (≥-2 z scores)	292	73.4
**Prevalence of stunting by gender**		
**Males N=197**		
**Overall stunting (<-2 z scores)**	**56**	**28.4**
Moderate stunting (<-2 to ≥-3)	45	22.4
Severe stunting (<-3 z scores)	10	5.1
**Females N=201**		
**Overall stunting (<-2 z scores)**	**50**	**24.9**
Moderate stunting (<-2 to ≥-3)	45	22.6
Severe stunting (<-3 z scores)	5	2.5

### Knowledge of stunting among caregivers

Over two-thirds of the caregivers (69.6%) had heard of stunting. Among mothers who had heard of stunting, 65.7 percent and 28.5% said a child is stunted if their height was shorter than average, and if the child's height did not match the child's age, respectively. Concerning the causes of stunting, close to two-thirds (65.3%) mentioned poor feeding or inadequate food intake. A considerable proportion of mothers (29.6 percent) said they did not know the causes of stunting. Regarding the consequences of stunting, 40.4% of mothers indicated that the child gets sick easily, and a similar proportion of mothers (39%) said they did not know. During the FGDs, mothers were asked what stunting meant to them, its causes and effects. The most common answer was “*Stunting means the child is not growing”*. When probed further, most caregivers said that growth meant increasing size and adding weight.

### Caregiver's knowledge of stunting as a predictor of stunting in children

No significant association was found between caregiver's knowledge of stunting and stunting in children below the age of five in Kitui County (table 3).

**Table 3:** Logistic regression analysis of caregiver's knowledge of stunting and stunting in children

Variable	Stunted HAZ<-2SD n (%)	Not stunted HAZ≥-2SD n (%)	Crude Odds Ratio (95% CI)	p-value
**Knowledge of stunting (N=277)**				
No knowledge	2 (18.2)	9 (81.8)	0.768 (0.159-3.721)	0.743
Little knowledge	20 (27.0)	54 (73.0)	1.383 (0.712-2.685)	0.446
Moderate knowledge	13 (27.7)	34 (72.3)	1.322 (0.630-2.775)	0.461
High knowledge	35 (22.4)	121 (77.6)	Reference	

### Child feeding practices

More than half of the babies (59%) began breastfeeding immediately after birth. Nearly one-third (30.1 percent) of the babies were given complementary foods before the recommended six months. A greater proportion (78.9%) of all surveyed children 6 to 23 months were still breastfeeding at one year. The results of the qualitative analysis revealed that child feeding practices were not optimal. A mother explained: “*Because I delivered through caesarean section, I could not breastfeed immediately, so I waited for one day to start breastfeeding”*. Another participant said: “*Up to six months I was breastfeeding but I also gave my child cow's milk that is boiled well”*.

### Child feeding practices associated with stunting

Children who were fed on complementary foods before 6 months had a 2.6 times greater chance of being stunted compared to those initiated to complementary foods at six months. Table 4 outlines the findings.

**Table 4:** Logistic regression analysis of child feeding practices and stunting

Variable	Stunted HAZ<-2SD n (%)	Not stunted HAZ≥-2SD n (%)	Crude Odds Ratio (95% CI)	p-value
**Initiation to breastfeeding (N=156)**				
< 1 hour	19 (20.7)	73 (79.3)	Reference	
≥ 1 hour	20 (31.2)	44 (68.8)	1.746 (0.841-3.627)	0.135
**Introduction of complementary foods (N=156)**				
< 6 months	18 (38.3)	29 (61.7)	2.601 (1.221-5.543)	0.013*
≥ 6 months	21 (19.3)	88 (80.7)	Reference	
**Is child still breastfeeding (N=156)**				
No	10 (37.0)	17 (63.0)	2.028 (0.838-4.909)	0.117
Yes	29 (22.5)	100 (77.5)	Reference	

* **Significant association between variables (p-value < 0.05)**

### Morbidity status of children

The morbidity rate in the two weeks before the survey stood at 42.5 percent. The leading causes of morbidity in children were fever (41.7%), cough (26.0%), and diarrhoea (19.7%). The majority of caregivers (62.3%) did not seek treatment for their sick children. One respondent made this observation: “*When my child is sick, I go to a traditional healer and later visit the hospital for treatment”*.

### Association between the morbidity status and stunting

There was no significant association between the morbidity status and stunting as shown in table 5.

**Table 5:** Logistic regression analysis of morbidity status and stunting

Variable	Stunted HAZ<-2SD n (%)	Not stunted HAZ≥-2SD n (%)	Crude Odds Ratio (95% CI)	p-value
**Illness in the last 2 weeks (N=398)**				
No	60 (26.2)	169 (73.8)	1.00 (reference)	
Yes	46 (27.2)	123 (72.8)	1.053 (0.672-1.650)	0.820

### Environmental characteristics of surveyed households

The primary source of drinking water for the surveyed families was rivers (43.5%). Greater than half of the respondents (54%) did not treat their drinking water. Handwashing with soap was practiced by a greater majority of households (94.7%). A large majority of households (97.5%) had latrines within their home compounds. Qualitative data identified constraints accessing water and sanitation. A mother commented: “*I'm not able to purchase clean water, so instead I get it from the river which is far and the water is dirty”*. Another participant added: “I *have a problem because we constructed a temporary latrine which can easily collapse and that will force us to share a latrine with our neighbors or go to the bush”*.

### Influence of environmental factors on stunting

According to the analysis in table 6, consumption of water from unimproved water sources raised the likelihood of stunting by 1.8 times when compared with consumption of water from improved sources. Further, babies from families with access to a latrine/toilet had 85.3% lower risk of stunting than children from homes with no access to a latrine.

**Table 6:** Logistic regression of the association between environmental factors and stunting in children 6-59 months in Kitui County

Variable	Stunted HAZ<-2SD n (%)	Not stunted HAZ≥-2SD n (%)	Crude Odds Ratio (95% CI)	p-value
**Sources of drinking water (N=398)**				
Unimproved drinking water source	77 (30.4)	176 (69.6)	1.750 (1.075-2.848)	0.024*
Improved drinking water source	29 (20.0)	116 (80.0)	Reference	
**Treatment of drinking water (N=398)**				
Not treating drinking water	60 (27.9)	155 (72.1)	1.153 (0.737-1.804)	0.533
Treating drinking water	46 (25.1)	137 (74.9)	Reference	
**Handwashing with soap (N=398)**				
Hand washing with no soap	7 (33.3)	14 (66.7)	1.404 (0.551-3.579)	0.477
Handwashing with soap	99 (26.3)	278 (73.7)	Reference	
**Household access to latrine/toilet (N=398)**				
No	7 (70.0)	3 (30.0)	Reference	
Yes	99 (25.5)	289 (74.5)	0.147 (0.037-0.579)	0.006*

### Multivariable logistic regression analyses on factors associated with stunting in children aged 6 to 59 months in Kitui County

Significant variables (p<0.05) in the bivariate logistic regression analyses were added to the multivariable logistic regression model. These included child's birth weight, mother's education level, mother's MUAC, household income, number of children below five in the family, household source of drinking water, and household access to latrine/toilet. The backward stepwise method of selecting variables was used to remove predictors that did not significantly contribute to the model. Only four of the seven investigated covariates remained significant in the multivariable logistic regression analysis. Results are reported with the Adjusted Odds Ratios (AOR) and their 95% CI. Thetatistical significance level was at a p-value less than 0.05. A child's birth weight and stunting were significantly correlated. Children with a birth weight <2500 grams had double the risk of stunting when compared to children with a birth weight of 2500 grams or more (AOR = 2.302; 95% CI: 1.027-6.150; p: 0.043). The findings also revealed a statistically significant relationship between a mother's education and stunting. Children of mothers with secondary education were 5.4 times more likely to be stunted compared to children of mothers with post-secondary education (AOR = 5.404; 95% CI: 1.607-18.173; p: 0.006). The risk of stunting in children of mothers with MUAC values < 23 cm was 2.8 times greater than in children whose mothers had MUAC values ≥ 23 cm (AOR = 2.845; 95% CI: 1.125-7.192; p:0.012). Children from households with access to latrines/toilets were 93.5% less likely to become stunted than children from homes that did not have access to latrines/toilets (AOR = 0.008; 95% CI: 0.009-0.561; p: 0.013).

## Discussion

The rate of stunting in Kitui Central Sub-County was high at 26.6%^17^. The current stunting rate was comparable to a previous study in Kitui County's Mwingi Central Sub-county, which found a prevalence of 27.9%^18^. Among the sociodemographic factors, mother's education level was statistically related to stunting. The stunting odds were greater in children of mothers with secondary education than in children of mothers with college/university education. This result was similar to a previous survey in Uganda^19^. A child's birth weight and stunting were significantly correlated. This finding concurred with studies in other parts of the world^20^,^21^,^22^. The maternal nutrition status was proven to significantly raise the risk of stunting. This finding was consistent with recent research in Cambodia and Bangladesh where babies delivered by mothers with low MUAC had increased risk of stunting^23^,^24^. However, the findings are contradicted by a survey in Ethiopia which found that a mother's MUAC was not significantly related to stunting^25^. The majority of caregivers (69.6%) had general knowledge of stunting, but some thought of stunting as failure to gain height and weight. Additionally, a significant proportion of caregivers (39%) had no knowledge of the effects of stunting. This finding was similar to another study conducted in Kenya which showed that mothers had a general understanding of stunting but failed to differentiate it from other forms of malnutrition^4^. Although this survey noted a high morbidity rate of 42.5%, illness was not significantly related to stunting. In Bangladesh, Alam et al.^12^ also found no association between morbidity and stunting. However, evidence from various studies has established the link between illness and stunting. Studies in Uganda^27^, Tanzania 26, and Ethiopia 28 highlighted morbidity as a major risk factor for stunting. Premised on these studies, illness may cause poor dietary intake, impaired absorption, and increased nutrient requirements, leaving the body deficient of nutrients for growth. Access to latrine/toilet was significantly related to stunting. Children from homes that had a latrine/toilet were 93.5% less likely to become stunted than children from homes without a latrine/toilet. Similar observations have been made in other studies done around the globe. In rural India, Rah et al.^29^ reported that access to sanitation facilities was linked to lower odds of stunting in children when compared to homes that practiced open defecation. Evidence suggests that undernutrition is caused by environment linked enteropathy induced by the ingestion of fecal bacteria by young children who live in unsanitary environments^30^.

## Conclusion

Stunting prevalence in Kitui County stood at 26.6% and is considered high based on the WHO chronic malnutrition threshold. The sociodemographic factors that had a significant association with stunting include child birth weight, mother's education level, and mother's nutrition status. Most caregivers in Kitui County had general knowledge of stunting. The morbidity status of children in Kitui County had no significant association with linear growth of children. Considering environmental factors, access to latrine/toilet was significantly related to stunting. The county government should implement a community literacy program to educate caregivers on stunting, intensify efforts for stunting prevention, enhance policies for women and girls education, enhance baby friendly community initiatives, and continue the current efforts to improve access to sanitation.

## Limitations of the study

This survey relied on self-reporting by caregivers who were interviewed and this may have affected the accuracy of data due to recall bias. The study was conducted in Kitui Central Sub-County and this limits the ability to generalize findings to the whole of Kitui County.

## Figures and Tables

**Table 7:** Multivariable analyses of variables associated with stunting in children aged 6 to 59 months in Kitui County

Variable	HAZ<-2SD (Stunted) n (%)	HAZ=-2SD (Not stunted) n (%)	Crude Odds Ratio (95% CI)	p-value	Adjusted Odds Ratio (95% CI)	p-value
**Child's birth weight (N=398)**						
<2500gms	22 (44.0)	28 (56.0)	2.469 (1.342-4.545)	0.004*	2.302 (1.027-5.160)	0.043*
=2500gms	87 (24.1)	264 (75.9)	1.00 (reference)			
**Mother's education level (N=398)**						
Secondary	97 (30.0)	226 (70.0)	3.147 (1.508-6.570)	0.002*	5.404 (1.607-18.173)	0.006*
Post-secondary	9 (12.0)	66 (88.0)	Reference			
**Mother's nutrition status (N=293)**						
<23 cm	11 (50.0)	11 (50.0)	3.106 (1.288-7.493)	0.012*	2.845 (1.125-7.192)	0.027*
=23cm	66 (24.4)	205 (75.6)	Reference			
**Children under 5 in HH (N=398)**						
1	68 (23.7)	219 (76.3)	Reference		0.593 (0.328-1.071)	
>1	38 (34.2)	73 (65.8)	1.676 (1.040-2.702)	0.034*		0.083
**Household income (N=398)**						
<10,000	73 (32.6)	151 (67.4)	5.125 (1.965-13.364)	0.001*	2.258 (0.678-7.527)	0.185
10,000-19,000	28 (24.1)	88 (75.9)	3.373 (1.227-9.267)	0.018*	1.919 (0.545-6.758)	0.310
=20,000	5 (8.6)	53 (91.4)	Reference			
**Sources of drinking water (N=398)**						
Unimproved drinking water source	77 (30.4)	176 (69.6)	1.750 (1.075-2.848)	0.024 *	1.099 (0.572-2.111)	0.777
Improved drinking water source	29 (20.0)	116 (80.0)	Reference			
**Household access to latrine/toilet (N=398)**						
No	7 (70.0)	3 (30.0)	Reference			
Yes	99 (25.5)	289 (74.5)	0.147 (0.037-0.579)	0.006*	**0.065 (0.008-0.561)**	0.013*

## Data Availability

The corresponding author may make the dataset available subject to ethical restriction.
